# *N*′-[1-(2-Hydroxy­phen­yl)ethyl­idene]benzene­sulfonohydrazide

**DOI:** 10.1107/S1600536808010970

**Published:** 2008-04-26

**Authors:** Xi-Shi Tai, Jun Xu, Yi-Min Feng, Zu-Pei Liang

**Affiliations:** a Department of Chemistry and Chemical Engineering, Weifang University, Weifang 261061, People’s Republic of China; b Weifang Institute of Supervision and Inspection of product Quality, Weifang 261061, People’s Republic of China

## Abstract

In the title compound, C_14_H_14_N_2_O_3_S, the conformation is stabilized by an intra­moleclar O—H⋯N hydrogen bond and the dihedral angle between the aromatic ring planes is 79.55 (18)°. In the crystal structure, inter­molecular N—H⋯O hydrogen bonds lead to [100] chains of mol­ecules.

## Related literature

For related literature, see: Tai *et al.* (2003 ▶).
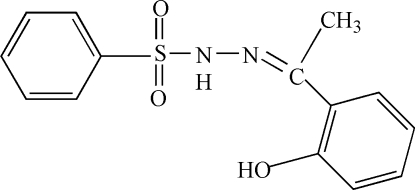


## Experimental

### 

#### Crystal data

C_14_H_14_N_2_O_3_S
*M* *_r_* = 290.33
Monoclinic, 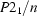

*a* = 5.2435 (9) Å
*b* = 13.2515 (18) Å
*c* = 20.375 (2) Å
β = 90.531 (2)°
*V* = 1415.7 (3) Å^3^
*Z* = 4
Mo *K*α radiation
μ = 0.24 mm^−1^
*T* = 298 (2) K
0.50 × 0.40 × 0.37 mm

#### Data collection

Bruker SMART CCD diffractometer
Absorption correction: multi-scan (*SADABS*; Bruker, 2000 ▶) *T*_min_ = 0.891, *T*_max_ = 0.918
7165 measured reflections
2484 independent reflections
1755 reflections with *I* > 2σ(*I*)
*R*_int_ = 0.062

#### Refinement

*R*[*F* ^2^ > 2σ(*F* ^2^)] = 0.047
*wR*(*F* ^2^) = 0.131
*S* = 1.04
2484 reflections
182 parameters
H-atom parameters constrained
Δρ_max_ = 0.26 e Å^−3^
Δρ_min_ = −0.26 e Å^−3^

### 

Data collection: *SMART* (Bruker, 2000 ▶); cell refinement: *SAINT* (Bruker, 2000 ▶); data reduction: *SAINT*; program(s) used to solve structure: *SHELXS97* (Sheldrick, 2008 ▶); program(s) used to refine structure: *SHELXL97* (Sheldrick, 2008 ▶); molecular graphics: *SHELXTL* (Sheldrick, 2008 ▶); software used to prepare material for publication: *SHELXTL*.

## Supplementary Material

Crystal structure: contains datablocks global, I. DOI: 10.1107/S1600536808010970/hb2723sup1.cif

Structure factors: contains datablocks I. DOI: 10.1107/S1600536808010970/hb2723Isup2.hkl

Additional supplementary materials:  crystallographic information; 3D view; checkCIF report

## Figures and Tables

**Table 1:** Hydrogen-bond geometry (Å, °)

*D*—H⋯*A*	*D*—H	H⋯*A*	*D*⋯*A*	*D*—H⋯*A*
O3—H3⋯N2	0.82	1.85	2.561 (3)	145
N1—H1⋯O2^i^	0.90	2.20	3.093 (3)	174

Symmetry code: (i) 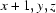.

**Fig. 1.**
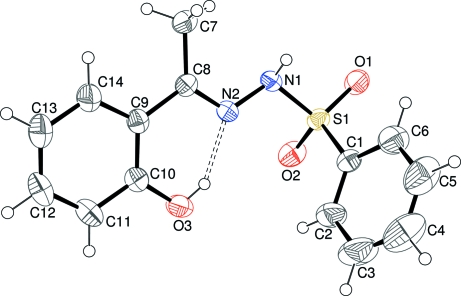
The molecular structure of (I) showing 40% displacement ellipsoids (arbitrary spheres for the H atoms). The hydrogen bond is indicated by a double-dashed line.

## supplementary crystallographic information

### Comment

As part of our ongoing studies of the coordination chemistry of aroylhydrazones as potential ligands (Tai *et al.*, 2003), we now report the synthesis and structure of the title compound, (I), (Fig. 1).

The molecular conformation is stabilised by an intramoleclar O-H···N hydrogen bond (Table 1) and the dihedral angle between the aromatic ring planes is 79.55 (18)°. In the crystal, an intermolecular N-H···O hydrogen bond lead to [100] chains of molecules.

### Experimental

3 mmol of 2'-hydroxyacetophenone (3 mmol) was added to a solution of benzenesulfonyl hydrazide (3 mmol) in 10 ml of 95% ethanol. The mixture was continuously stirred for 3 h at refluxing temperature, evaporating some ethanol, then, upon cooling, the solid product was collected by filtration and dried *in vacuo* (yield 68%). Colourless blocks of (I) were obtained by evaporation from a methanol solution after 3 days.

### Refinement

The H atoms were placed geometrically (C—H = 0.93–0.96 Å, O—H = 0.82Å, N—H = 0.90 Å) and refined as riding with *U*_iso_(H) = 1.2*U*_eq_(carrier) or 1.5*U*_eq_(methyl C).

### Crystal data

**Table:**

C_14_H_14_N_2_O_3_S	*F*_000_ = 608
*M**_r_* = 290.33	*D*_x_ = 1.362 Mg m^−^^3^
Monoclinic, *P*2_1_/*n*	Mo *K*α radiation λ = 0.71073 Å
Hall symbol: -P 2yn	Cell parameters from 2187 reflections
*a* = 5.2435 (9) Å	θ = 2.5–26.2º
*b* = 13.2515 (18) Å	µ = 0.24 mm^−^^1^
*c* = 20.375 (2) Å	*T* = 298 (2) K
β = 90.531 (2)º	Block, colourless
*V* = 1415.7 (3) Å^3^	0.50 × 0.40 × 0.37 mm
*Z* = 4	

### Data collection

**Table:**

Bruker SMART CCD diffractometer	2484 independent reflections
Radiation source: fine-focus sealed tube	1755 reflections with *I* > 2σ(*I*)
Monochromator: graphite	*R*_int_ = 0.062
*T* = 298(2) K	θ_max_ = 25.0º
ω scans	θ_min_ = 1.8º
Absorption correction: multi-scan(SADABS; Bruker, 2000)	*h* = −6→6
*T*_min_ = 0.891, *T*_max_ = 0.918	*k* = −15→15
7165 measured reflections	*l* = −20→24

### Refinement

**Table:**

Refinement on *F*^2^	Hydrogen site location: inferred from neighbouring sites
Least-squares matrix: full	H-atom parameters constrained
*R*[*F*^2^ > 2σ(*F*^2^)] = 0.047	*w* = 1/[σ^2^(*F*_o_^2^) + (0.0428*P*)^2^ + 0.8643*P*] where *P* = (*F*_o_^2^ + 2*F*_c_^2^)/3
*wR*(*F*^2^) = 0.131	(Δ/σ)_max_ = 0.001
*S* = 1.04	Δρ_max_ = 0.26 e Å^−^^3^
2484 reflections	Δρ_min_ = −0.26 e Å^−^^3^
182 parameters	Extinction correction: SHELXL97 (Sheldrick, 2008), Fc^*^=kFc[1+0.001xFc^2^λ^3^/sin(2θ)]^-1/4^
Primary atom site location: structure-invariant direct methods	Extinction coefficient: 0.018 (2)
Secondary atom site location: difference Fourier map	

### Special details

**Table:**

Geometry. All e.s.d.'s (except the e.s.d. in the dihedral angle between two l.s. planes) are estimated using the full covariance matrix. The cell e.s.d.'s are taken into account individually in the estimation of e.s.d.'s in distances, angles and torsion angles; correlations between e.s.d.'s in cell parameters are only used when they are defined by crystal symmetry. An approximate (isotropic) treatment of cell e.s.d.'s is used for estimating e.s.d.'s involving l.s. planes.
Refinement. Refinement of *F*^2^ against ALL reflections. The weighted *R*-factor *wR* and goodness of fit *S* are based on *F*^2^, conventional *R*-factors *R* are based on *F*, with *F* set to zero for negative *F*^2^. The threshold expression of *F*^2^ > σ(*F*^2^) is used only for calculating *R*-factors(gt) *etc*. and is not relevant to the choice of reflections for refinement. *R*-factors based on *F*^2^ are statistically about twice as large as those based on *F*, and *R*- factors based on ALL data will be even larger.

### Fractional atomic coordinates and isotropic or equivalent isotropic displacement parameters (Å^2^)

**Table:**

	*x*	*y*	*z*	*U*_iso_*/*U*_eq_	
N1	0.3921 (4)	0.13317 (15)	0.94596 (10)	0.0397 (6)	
H1	0.5569	0.1345	0.9587	0.048*	
N2	0.3516 (4)	0.21056 (16)	0.90070 (10)	0.0378 (5)	
O1	0.2888 (4)	0.06000 (14)	1.05125 (9)	0.0589 (6)	
O2	−0.0427 (4)	0.15363 (16)	0.98874 (10)	0.0560 (6)	
O3	0.0643 (4)	0.35673 (15)	0.86405 (10)	0.0590 (6)	
H3	0.1123	0.3112	0.8884	0.089*	
S1	0.21361 (14)	0.14329 (5)	1.01133 (3)	0.0403 (3)	
C1	0.2935 (5)	0.2560 (2)	1.05229 (13)	0.0414 (7)	
C2	0.1465 (7)	0.3404 (2)	1.04223 (18)	0.0659 (10)	
H2	0.0090	0.3388	1.0131	0.079*	
C3	0.2063 (10)	0.4282 (3)	1.0763 (2)	0.0931 (14)	
H3A	0.1108	0.4864	1.0694	0.112*	
C4	0.4057 (10)	0.4288 (4)	1.1200 (3)	0.1013 (16)	
H4	0.4428	0.4871	1.1436	0.122*	
C5	0.5504 (8)	0.3446 (4)	1.1293 (2)	0.0922 (14)	
H5	0.6876	0.3463	1.1586	0.111*	
C6	0.4952 (6)	0.2567 (3)	1.09574 (17)	0.0651 (9)	
H6	0.5928	0.1990	1.1024	0.078*	
C7	0.6988 (6)	0.1407 (2)	0.83455 (14)	0.0472 (7)	
H7A	0.8593	0.1693	0.8479	0.071*	
H7B	0.7018	0.1265	0.7884	0.071*	
H7C	0.6699	0.0793	0.8584	0.071*	
C8	0.4892 (5)	0.21397 (19)	0.84856 (12)	0.0357 (6)	
C9	0.4232 (5)	0.29502 (19)	0.80152 (12)	0.0381 (6)	
C10	0.2176 (5)	0.3608 (2)	0.81077 (13)	0.0421 (7)	
C11	0.1611 (6)	0.4351 (2)	0.76493 (15)	0.0553 (8)	
H11	0.0230	0.4779	0.7716	0.066*	
C12	0.3072 (7)	0.4462 (3)	0.70987 (16)	0.0619 (9)	
H12	0.2675	0.4960	0.6793	0.074*	
C13	0.5109 (7)	0.3840 (3)	0.70007 (15)	0.0631 (9)	
H13	0.6118	0.3920	0.6631	0.076*	
C14	0.5665 (6)	0.3096 (2)	0.74492 (14)	0.0524 (8)	
H14	0.7047	0.2673	0.7373	0.063*	

### Atomic displacement parameters (Å^2^)

**Table:**

	*U*^11^	*U*^22^	*U*^33^	*U*^12^	*U*^13^	*U*^23^
N1	0.0424 (14)	0.0379 (12)	0.0388 (12)	0.0084 (10)	0.0018 (10)	0.0056 (10)
N2	0.0423 (13)	0.0336 (12)	0.0374 (12)	0.0046 (10)	−0.0001 (10)	0.0029 (10)
O1	0.0933 (17)	0.0375 (11)	0.0461 (12)	0.0088 (11)	0.0154 (11)	0.0074 (9)
O2	0.0413 (13)	0.0634 (14)	0.0636 (13)	−0.0068 (10)	0.0049 (10)	−0.0136 (11)
O3	0.0633 (15)	0.0536 (13)	0.0605 (13)	0.0217 (11)	0.0148 (11)	0.0146 (10)
S1	0.0473 (5)	0.0335 (4)	0.0402 (4)	0.0002 (3)	0.0065 (3)	−0.0006 (3)
C1	0.0423 (17)	0.0383 (15)	0.0436 (16)	−0.0023 (12)	0.0051 (13)	−0.0047 (12)
C2	0.081 (3)	0.0445 (19)	0.073 (2)	0.0108 (17)	−0.0073 (19)	−0.0115 (16)
C3	0.123 (4)	0.046 (2)	0.111 (3)	0.009 (2)	0.011 (3)	−0.022 (2)
C4	0.102 (4)	0.084 (3)	0.118 (4)	−0.034 (3)	0.012 (3)	−0.050 (3)
C5	0.069 (3)	0.114 (4)	0.094 (3)	−0.019 (3)	−0.012 (2)	−0.045 (3)
C6	0.048 (2)	0.074 (2)	0.073 (2)	0.0026 (17)	−0.0069 (18)	−0.0137 (18)
C7	0.0505 (19)	0.0457 (16)	0.0457 (16)	0.0071 (14)	0.0069 (14)	−0.0027 (13)
C8	0.0362 (15)	0.0342 (14)	0.0366 (14)	−0.0051 (12)	−0.0013 (12)	−0.0055 (11)
C9	0.0418 (17)	0.0361 (14)	0.0363 (14)	−0.0049 (12)	−0.0048 (12)	−0.0004 (11)
C10	0.0421 (17)	0.0400 (15)	0.0442 (16)	−0.0037 (13)	−0.0021 (13)	0.0025 (13)
C11	0.056 (2)	0.0466 (18)	0.063 (2)	0.0022 (15)	−0.0082 (16)	0.0154 (15)
C12	0.071 (2)	0.059 (2)	0.055 (2)	−0.0072 (18)	−0.0139 (18)	0.0214 (16)
C13	0.076 (3)	0.069 (2)	0.0450 (18)	−0.0073 (19)	0.0049 (17)	0.0140 (16)
C14	0.056 (2)	0.0562 (19)	0.0448 (17)	0.0011 (15)	0.0025 (15)	0.0065 (14)

### Geometric parameters (Å, °)

**Table:**

N1—N2	1.394 (3)	C5—H5	0.9300
N1—S1	1.641 (2)	C6—H6	0.9300
N1—H1	0.9000	C7—C8	1.496 (4)
N2—C8	1.291 (3)	C7—H7A	0.9600
O1—S1	1.4246 (19)	C7—H7B	0.9600
O2—S1	1.423 (2)	C7—H7C	0.9600
O3—C10	1.358 (3)	C8—C9	1.478 (4)
O3—H3	0.8200	C9—C14	1.395 (4)
S1—C1	1.760 (3)	C9—C10	1.401 (4)
C1—C2	1.372 (4)	C10—C11	1.387 (4)
C1—C6	1.373 (4)	C11—C12	1.372 (4)
C2—C3	1.390 (5)	C11—H11	0.9300
C2—H2	0.9300	C12—C13	1.366 (5)
C3—C4	1.367 (6)	C12—H12	0.9300
C3—H3A	0.9300	C13—C14	1.374 (4)
C4—C5	1.361 (6)	C13—H13	0.9300
C4—H4	0.9300	C14—H14	0.9300
C5—C6	1.380 (5)		
			
N2—N1—S1	113.09 (16)	C5—C6—H6	120.6
N2—N1—H1	108.5	C8—C7—H7A	109.5
S1—N1—H1	108.5	C8—C7—H7B	109.5
C8—N2—N1	119.2 (2)	H7A—C7—H7B	109.5
C10—O3—H3	109.5	C8—C7—H7C	109.5
O2—S1—O1	120.99 (14)	H7A—C7—H7C	109.5
O2—S1—N1	106.82 (12)	H7B—C7—H7C	109.5
O1—S1—N1	104.08 (11)	N2—C8—C9	115.5 (2)
O2—S1—C1	106.95 (13)	N2—C8—C7	123.5 (2)
O1—S1—C1	108.88 (13)	C9—C8—C7	121.0 (2)
N1—S1—C1	108.63 (12)	C14—C9—C10	116.5 (2)
C2—C1—C6	121.3 (3)	C14—C9—C8	120.8 (2)
C2—C1—S1	119.3 (2)	C10—C9—C8	122.7 (2)
C6—C1—S1	119.3 (2)	O3—C10—C11	116.3 (3)
C1—C2—C3	118.9 (4)	O3—C10—C9	123.0 (2)
C1—C2—H2	120.5	C11—C10—C9	120.7 (3)
C3—C2—H2	120.5	C12—C11—C10	120.6 (3)
C4—C3—C2	119.9 (4)	C12—C11—H11	119.7
C4—C3—H3A	120.1	C10—C11—H11	119.7
C2—C3—H3A	120.1	C13—C12—C11	119.9 (3)
C5—C4—C3	120.5 (4)	C13—C12—H12	120.1
C5—C4—H4	119.7	C11—C12—H12	120.1
C3—C4—H4	119.7	C12—C13—C14	119.8 (3)
C4—C5—C6	120.6 (4)	C12—C13—H13	120.1
C4—C5—H5	119.7	C14—C13—H13	120.1
C6—C5—H5	119.7	C13—C14—C9	122.4 (3)
C1—C6—C5	118.8 (4)	C13—C14—H14	118.8
C1—C6—H6	120.6	C9—C14—H14	118.8
			
S1—N1—N2—C8	176.68 (19)	N1—N2—C8—C9	176.6 (2)
N2—N1—S1—O2	52.5 (2)	N1—N2—C8—C7	−2.1 (4)
N2—N1—S1—O1	−178.43 (18)	N2—C8—C9—C14	176.8 (2)
N2—N1—S1—C1	−62.5 (2)	C7—C8—C9—C14	−4.5 (4)
O2—S1—C1—C2	−17.1 (3)	N2—C8—C9—C10	−3.1 (4)
O1—S1—C1—C2	−149.4 (3)	C7—C8—C9—C10	175.6 (3)
N1—S1—C1—C2	97.9 (3)	C14—C9—C10—O3	−178.9 (3)
O2—S1—C1—C6	160.2 (2)	C8—C9—C10—O3	1.0 (4)
O1—S1—C1—C6	27.9 (3)	C14—C9—C10—C11	0.8 (4)
N1—S1—C1—C6	−84.8 (3)	C8—C9—C10—C11	−179.3 (3)
C6—C1—C2—C3	0.8 (5)	O3—C10—C11—C12	179.1 (3)
S1—C1—C2—C3	178.0 (3)	C9—C10—C11—C12	−0.6 (5)
C1—C2—C3—C4	−1.3 (6)	C10—C11—C12—C13	−0.3 (5)
C2—C3—C4—C5	1.6 (7)	C11—C12—C13—C14	1.0 (5)
C3—C4—C5—C6	−1.3 (7)	C12—C13—C14—C9	−0.7 (5)
C2—C1—C6—C5	−0.5 (5)	C10—C9—C14—C13	−0.2 (4)
S1—C1—C6—C5	−177.7 (3)	C8—C9—C14—C13	180.0 (3)
C4—C5—C6—C1	0.7 (6)		

### Hydrogen-bond geometry (Å, °)

**Table:**

*D*—H···*A*	*D*—H	H···*A*	*D*···*A*	*D*—H···*A*
O3—H3···N2	0.82	1.85	2.561 (3)	145
N1—H1···O2^i^	0.90	2.20	3.093 (3)	174

Symmetry codes: (i) *x*+1, *y*, *z*.
